# Obese-associated gut microbes and derived phenolic metabolite as mediators of excessive motivation for food reward

**DOI:** 10.1186/s40168-023-01526-w

**Published:** 2023-04-28

**Authors:** Alice de Wouters d’Oplinter, Marko Verce, Sabrina J. P. Huwart, Jacob Lessard-Lord, Clara Depommier, Matthias Van Hul, Yves Desjardins, Patrice D. Cani, Amandine Everard

**Affiliations:** 1 Metabolism and Nutrition Research Group, Louvain Drug Research Institute, UCLouvain, Université catholique de Louvain, Brussels, Belgium; 2 Walloon Excellence in Life Sciences and BIOtechnology (WELBIO) department, WEL Research Institute (WELRI), avenue Pasteur, 6, 1300 Wavre, Belgium; 3 Institute of Nutrition and Functional Foods (INAF), Faculty of Agriculture and Food Sciences, Laval University, Québec, QC Canada; 4 Nutrition, Health and Society Centre (NUTRISS), INAF, Laval University, Québec, QC Canada; 5 Department of Plant Science, Faculty of Agriculture and Food Sciences, Laval University, Québec, QC Canada

## Abstract

**Background:**

Excessive hedonic consumption is one of the main drivers for weight gain. Identifying contributors of this dysregulation would help to tackle obesity. The gut microbiome is altered during obesity and regulates host metabolism including food intake.

**Results:**

By using fecal material transplantation (FMT) from lean or obese mice into recipient mice, we demonstrated that gut microbes play a role in the regulation of food reward (i.e., wanting and learning processes associated with hedonic food intake) and could be responsible for excessive motivation to obtain sucrose pellets and alterations in dopaminergic and opioid markers in reward-related brain areas. Through untargeted metabolomic approach, we identified the 3-(3’-hydroxyphenyl)propanoic acid (33HPP) as highly positively correlated with the motivation. By administrating 33HPP in mice, we revealed its effects on food reward.

**Conclusions:**

Our data suggest that targeting the gut microbiota and its metabolites would be an interesting therapeutic strategy for compulsive eating, preventing inappropriate hedonic food intake.

Video Abstract

**Supplementary Information:**

The online version contains supplementary material available at 10.1186/s40168-023-01526-w.

## Introduction

Inappropriate food behavior combined with an increased availability of energy-dense foods is an important contributor to the emerging worldwide obesity epidemic [1]. To maintain a stable body weight, energy intake and energy expenditure should be balanced. This process is regulated at the level of the hypothalamus and is referred to as the homeostatic regulation of food intake [2]. By opposition, the reward system is referred to as the non-homeostatic regulation of food intake and induces hedonic—or pleasure-related—consumption of foods enriched in fat and sugar. The pleasure is encoded by a mechanism linked to the stimulation of dopamine release from dopaminergic neurons in mesocorticolimbic area of the brain [3]. These dopaminergic neurons project from the ventral tegmental area (VTA) to the striatum, the nucleus accumbens (NAc), and the prefrontal cortex (PFC). Although dopamine is the main neurotransmitter of the reward system, other mediators such as opioids and endocannabinoids have also been involved in hedonic feeding [4, 5]. The food reward can be divided into three components: the liking, the wanting, and the learning [6]. The liking refers to the hedonic value attributed to food; the wanting to the motivational process to seek out and consume certain foods; and the learning to the reinforcing of conditioning behavior, associated with food intake stimulus [7, 8]. Food reward alterations are considered to be major drivers for body weight gain, promoting obesity by overriding the homeostatic regulation of food intake. Obesity itself is often characterized by inappropriate behavioral components of the food reward system, as well as by a hypofunctioning of the dopaminergic pathways, leading to overconsumption of food in an attempt to compensate for the lack of pleasure generated [9–12].

Both neuronal (i.e., the vagus nerve) and humoral pathways link the gut and brain to modulate homeostatic and non-homeostatic food intakes [13]. Importantly, these two types of food intake regulations can overlap. For example, key hormones mediating homeostatic food intake (leptin, insulin, ghrelin, and GLP-1) also influence reward responses [2, 14–16]. In obesity, the plasma concentrations of these mediators are altered, leading to a perturbation of the gut-brain axis and dysregulated food behavior [2].

The gut microbiota has emerged as a key player mediating the gut-brain axis by interfering with vagal and humoral pathways to modulate host metabolism and food intake [17–20]. Gut microbes are able to stimulate the production of satiety hormones (GLP-1 and PYY) through the production of short-chain fatty acids (SCFAs), and to interact with the vagus nerve [18, 21]. In obesity, the gut microbiota composition is unbalanced and contributes to reinforcing the impairment of the gut-brain axis. Although the role of the gut microbiota in homeostatic regulation of food intake is largely described and supported by strong literature, the causal role of the gut microbiota in non-homeostatic regulation of food intake is poorly understood. We previously demonstrated the proof-of-concept that gut microbes from obese mice are involved in alterations of the liking component of food intake during obesity. Indeed, fecal material transplantation (FMT) is able to reproduce hedonic alterations of obese donor mice in lean mice transplanted with the gut microbiota harvested from obese animals [22].

In this study, we aim to assess the role of gut microbes in the wanting and the learning components of food reward using operant wall test and conditioned place preference (CPP) test respectively, both of which are validated tools for behavioral analysis in rodents. We evaluated these components in lean and obese mice (i.e., donor mice) as well as in lean mice transplanted with the gut microbiota harvested from these lean and obese donors (i.e., gut microbiota recipient mice). Our second objective is to point out how microbiota from obese donors could impact the food reward system, and to identify new mediators involved in the gut-brain axis by using untargeted metabolomics analyses.

## Results

### FMT does not transfer obese phenotype (fat mass and body weight) into lean recipient mice

**Fig. 1:**
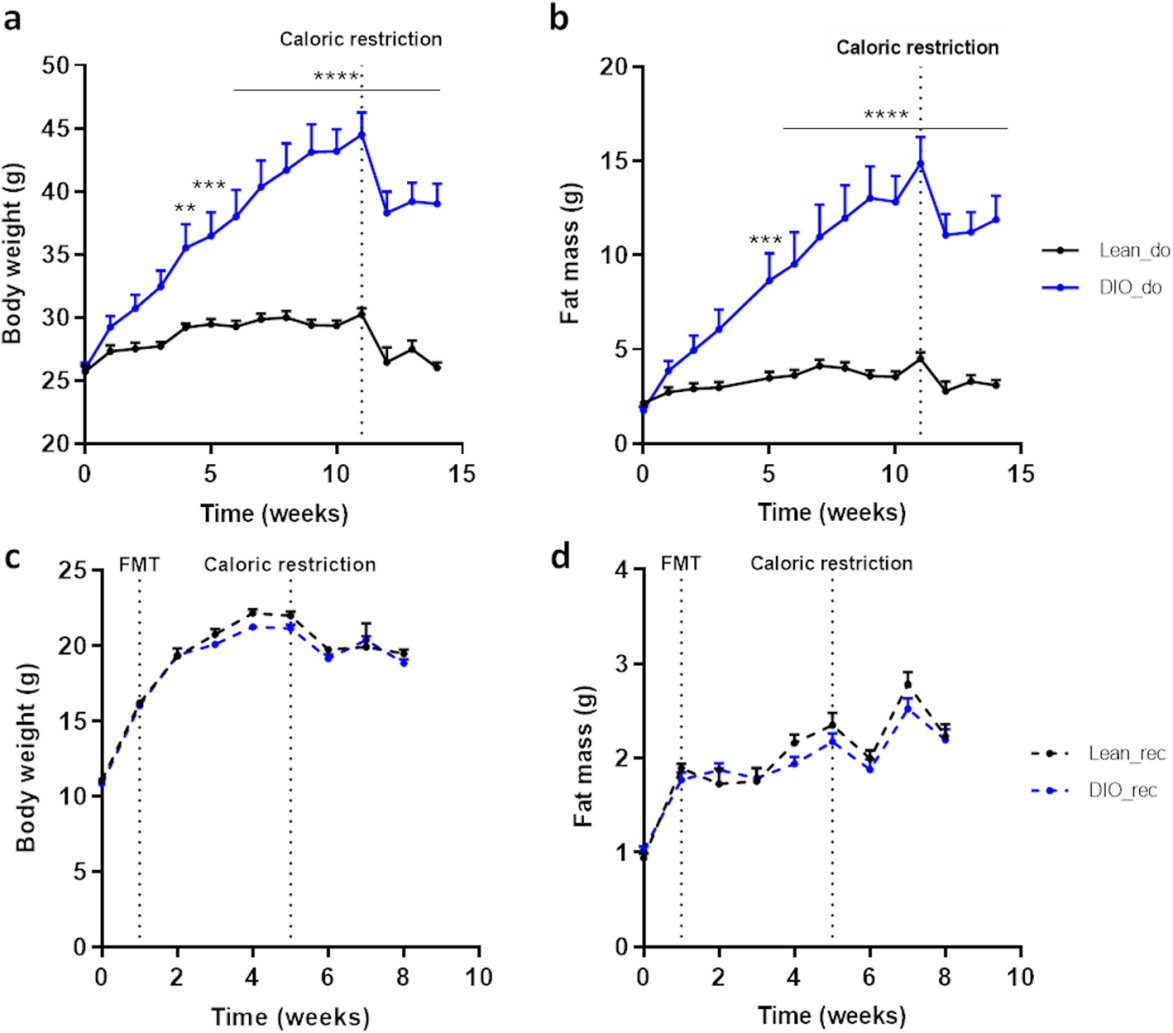
High-fat diet-induced obesity is not transferred through FMT in recipient mice.** a** Body weight and **b** fat mass evolutions (g) of lean (Lean_do) and diet-induced obese donor mice (DIO_do). **c** Body weight and **d** fat mass evolutions (g) of gut microbiota recipient mice from lean (Lean_rec) and diet-induced obese donor mice (DIO_rec). Data are shown as mean ± SEM (*n*=7-8/group). *p*-values were obtained after two-way ANOVA, followed by Bonferroni post hoc test. **: *p*-value ≤ 0.01; *** : *p*-value ≤ 0.001; **** : *p*-value ≤ 0.0001

We first validated the development of obesity in the cohort of donor mice: obese donors fed with high-fat diet (HFD) showed a higher body weight and fat mass compared to lean donors (Fig. 1a, b). For the food behavior tests, caloric restriction to reach 85% of initial body weight was required to potentiate the activation of the food reward system [23, 24]. However, the differences in body weight and fat mass were maintained all along the experiment and mice under HFD remained significantly heavier and fatter than mice under CT diet during the subsequent behavioral tests (Fig. 1a, b). We used the FMT protocol previously described to assess the role of the gut microbiota in the learning and motivational components of the food reward [22]. In brief, we sampled fecal samples from lean or obese mice and inoculated them by oral gavage into antibiotic-treated recipient mice. All recipient mice were kept under the same low-fat control diet. There was no difference of body weight (Fig. 1c) or fat mass (Fig. 1d) between gut microbiota recipient mice from obese and lean donors. As in our previous FMT experiment and many others in the literature, donor mice did not transfer their obese phenotype into recipient mice after FMT [22, 25].

### Lean or obese microbiota signatures are maintained after FMT

**Fig. 2:**
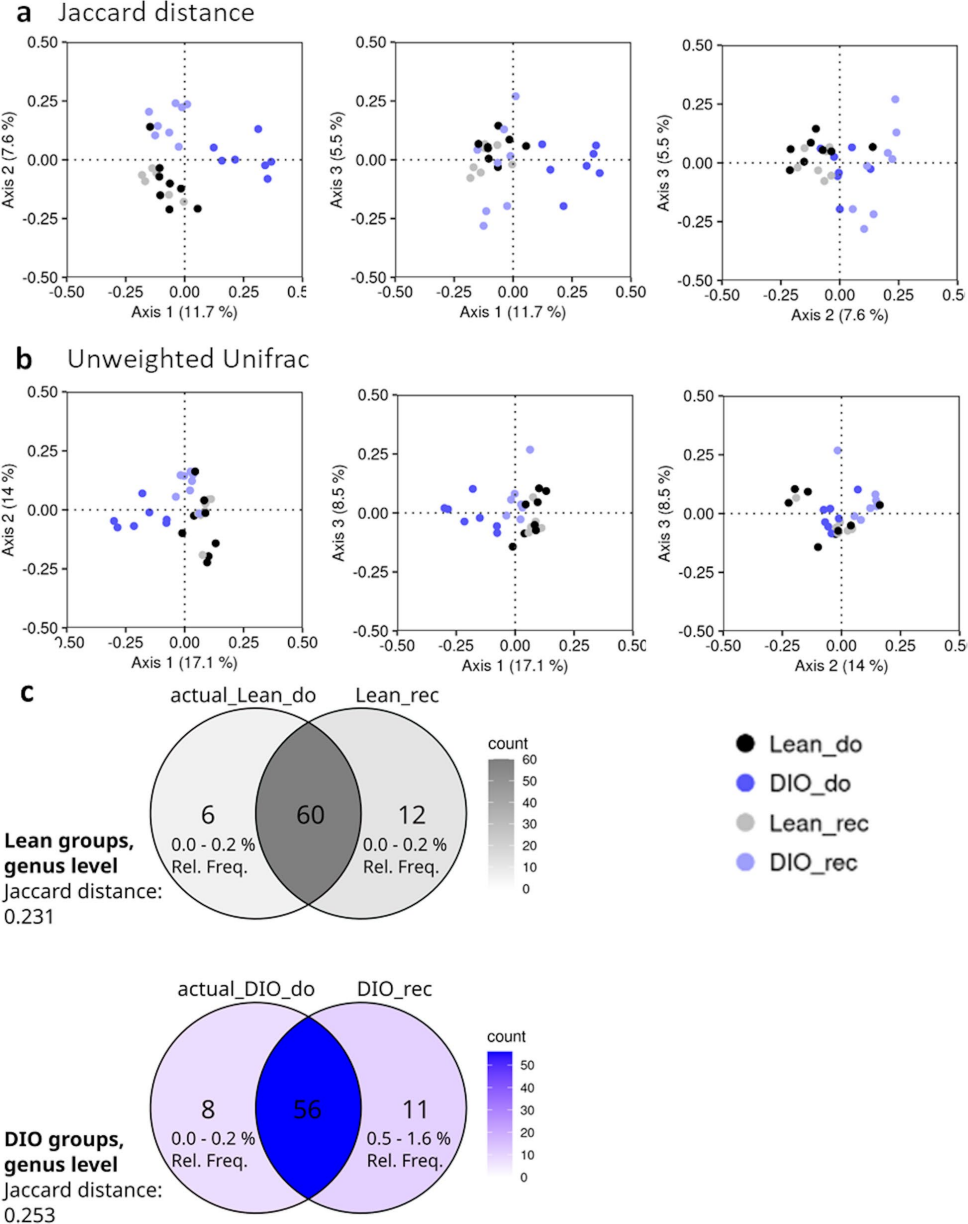
The majority of gut microbes from lean and obese donor mice engrafted recipient mice. Principal coordinates analysis (PCoA) for ASV-level data based on **a** the Jaccard distance and **b** the unweighted UniFrac. Each symbol representing a single sample is colored according to its group. **c** Venn diagrams depicting shared genera between lean (Lean_do) and diet-induced obese donor mice (DIO_do) and their respective gut microbiota recipient mice (Lean_rec and DIO_rec)

In order to assess the correct engraftment of the donor gut microbiota into recipient mice, we compared gut microbiota compositions of donor and recipient mice. First, β-diversity indices showed significant modifications of gut microbiota in obese mice (DIO_do) compared to control mice (Lean_do) as these samples clustered separately on the PCoA plots (Fig. 2a, b) and these differences were supported by PERMANOVA for Jaccard distance and unweighted UniFrac (*p* < 0.05). Moreover, as expected and according to the scientific literature, β-diversity indices showed two clusters differentiating between the two diets along the PCoA1 axis (Fig. 2a). Indeed, Lean_do, Lean_rec, and DIO_rec groups under control diet stuck apart from the DIO_do group under HFD.

Importantly, Lean_do and Lean_rec samples overlapped on the PCoA plots, suggesting an appropriate transfer of gut microbiome from Lean_do to Lean_rec (Fig. 2a, b). Significant differences between these two groups shown by PERMANOVA (*p* < 0.05) in the case of Jaccard distances were most likely due to a significant difference in dispersion (Fig. 2a, PERMDISP, *p* < 0.01). DIO_do and DIO_rec samples formed separate clusters on the PCoA plots, but DIO_rec samples were also separate from the Lean_do and Lean_rec samples, suggesting that even if the composition of DIO_rec mice was affected by the CT diet, the difference from the lean gut microbiota persisted (Fig. 2a, b).

Consistently, Venn diagrams demonstrate that 76.9% of genera were shared between lean donors and lean gut microbiota recipient mice; and 74.7% of genera were shared between obese donors and their recipient mice (Fig. 2c).

### Alterations in the hedonic and learning components of the food reward associated with obesity are partially transferred through FMT

**Fig. 3:**
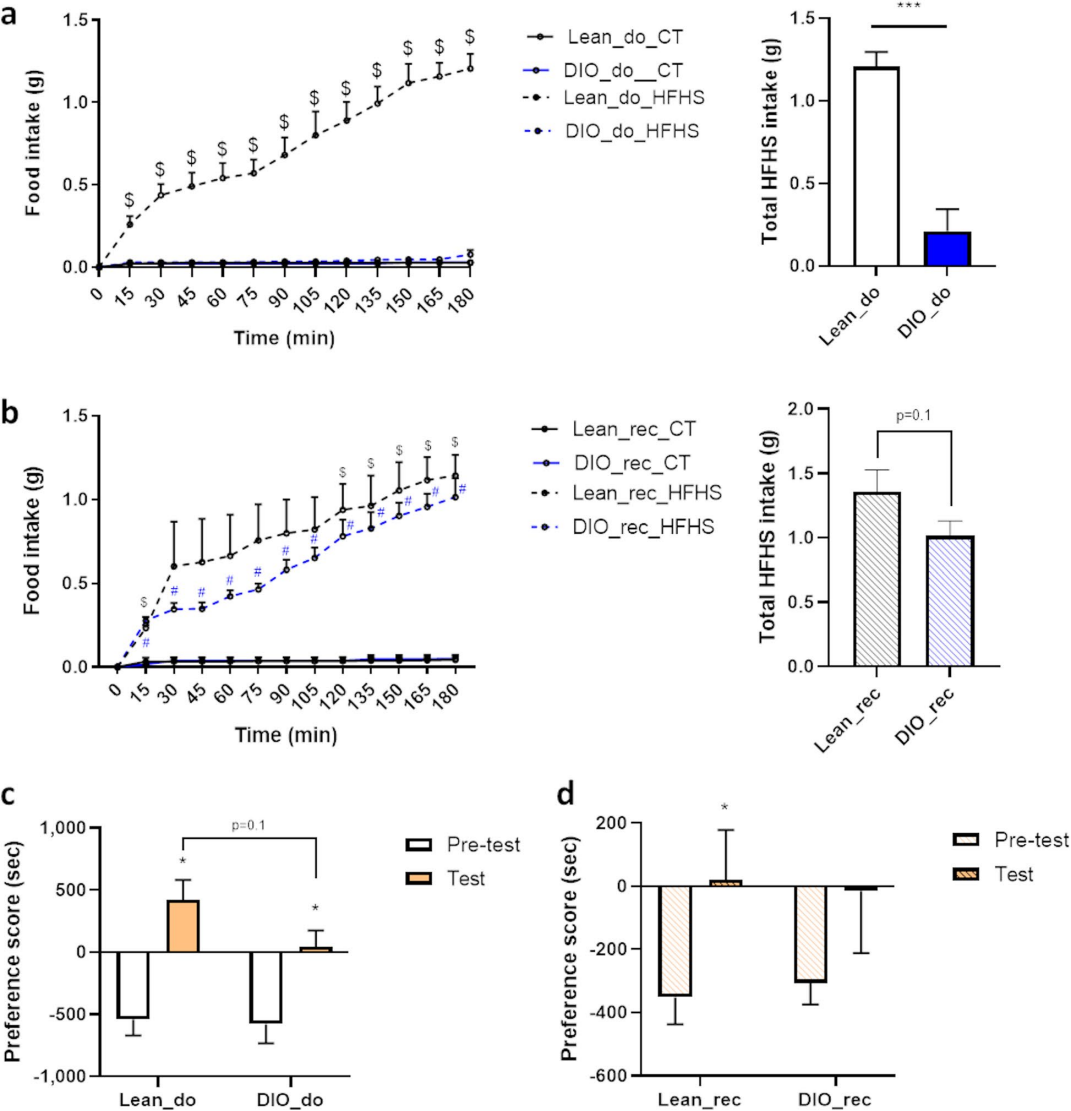
The alteration of the hedonic and learning components of food behavior associated with obesity is partially transferred by gut microbes. **a** Food preference test showing HFHS and CT intake every 15 min during 180 min of test and total HFHS intake after 180 min of test by lean (Lean_do) and DIO donor mice (DIO_do). **b** Food preference test showing HFHS and CT intake every 15 min during 180 min of test and total HFHS intake after 180 min of test by gut microbiota recipient mice from lean (Lean_rec) and diet-induced obese donor mice (DIO_rec). **c** Preference score of conditioned place preference based on the difference of time spent (s) in the palatable food-associated side *vs* the time spent in the neutral-associated side of the cage during the pre-test and the test by lean (Lean_do) or diet-induced obese donor mice (DIO_do). **d** Preference score of conditioned place preference based on the difference of time spent (s) in the palatable food-associated side vs the time spent in the neutral-associated side of the cage during the pre-test and the test by gut microbiota recipient mice from lean (Lean_rec) and diet-induced obese donor mice (DIO_rec). Data are shown as mean ± SEM (*n*=4–6/group). *p*-values were obtained after two-way ANOVA, followed by Bonferroni post hoc test (**a,b**) or after unpaired Student’s *t* test (**a,b**) or after paired Student’s *t* test (**c,d**). ^$^: *p*-value ≤ 0.05 between CT vs HFHS intake in Lean_do and Lean_rec group, ^#^: *p*-value ≤ 0.05 between CT vs HFHS intake in DIO_rec group, ***: *p*-value ≤ 0.001 between Lean_do group and DIO_do group *: *p*-value ≤ 0.05 between preference scores during test and pre-test

In order to evaluate the pleasure associated with palatable food intake independently of energy needs, we performed a food preference test by exposing mice to control diet (CT) and high-fat high-sugar diet (HFHS), 3 h a day during the inactive period and for 3 consecutive days. We analyzed control and HFHS intakes during the last day of the experiment to study the hedonic component of food intake independently of novelty. Lean mice eat significantly more HFHS than control diet, showing their tropism towards HFHS. However, obese mice did not show any tropism towards HFHS and eat significantly less HFHS than lean mice (Fig. 3a). These data revealed the alterations of the pleasure and reward process associated with palatable food intake during obesity as previously demonstrated [22]. Interestingly, even if both lean and obese gut microbiota recipient mice preferred HFHS diet to CT as they ate more HFHS than CT, obese gut microbiota recipient mice tend to eat less HFHS than lean gut microbiota recipient mice (*p*=0.1 DIO_rec versus Lean_rec) (Fig. 3b). Altogether these results confirmed a causal role of the gut microbiota in the hedonic food behavior alterations associated with obesity [22].

To investigate the roles of gut microbes in the learning component of food reward, we assessed the learning component of the food reward by CPP test in donor and recipient mice (Fig. 3c). The aim of this test is to evaluate to what extent mice could be conditioned to prefer a compartment with a food stimulus, even after the stimulus was removed. Here we aimed to increase the time spent by the mouse in one side of the cage after being restrained in this side during the training sessions with a palatable food pellet stimulating the reward system (Reese’s®). A pre-test is used to determine whether mice had a pre-existing preference for any of the compartments at baseline.

Both lean and obese donors spent more time in the compartment associated with palatable food during the test than during the pre-test, suggesting that they are both able to reverse their initial preference for one side of the cage after the training sessions (Fig. 3c). However, the learning component of the food reward is more efficient in lean mice than in obese mice. Indeed, the difference of time spent in the palatable food-associated compartment compared to neutral compartment tends to be lower in obese mice compared to lean mice (*p*=0.1, Fig. 3c).

Recipients of gut microbiota from lean donors also reversed their initial preference for one compartment and significantly increased the time spent in the palatable side during the test as compared to the pre-test (Fig. 3d). In opposition, even if they spent more time in the palatable associated compartment during the test, gut microbiota recipient mice from obese donors showed no significant difference in the preference score for the palatable side during the test as compared to the pre-test (Fig. 3d). The DIO_rec group failed to reverse their initial preference for one side of the cage, reflecting their inability to effectively associate the side of the cage with palatable food-induced pleasure. These results suggest that recipient mice of gut microbiota from obese donors have a dysregulated learning component of the food reward. Altogether, our data demonstrate that the alterations of the learning component associated with obesity are partially transferred through FMT between donor and recipient mice.

### Gut microbiota recipient mice from obese donors show excessive motivation for food reward

**Fig. 4:**
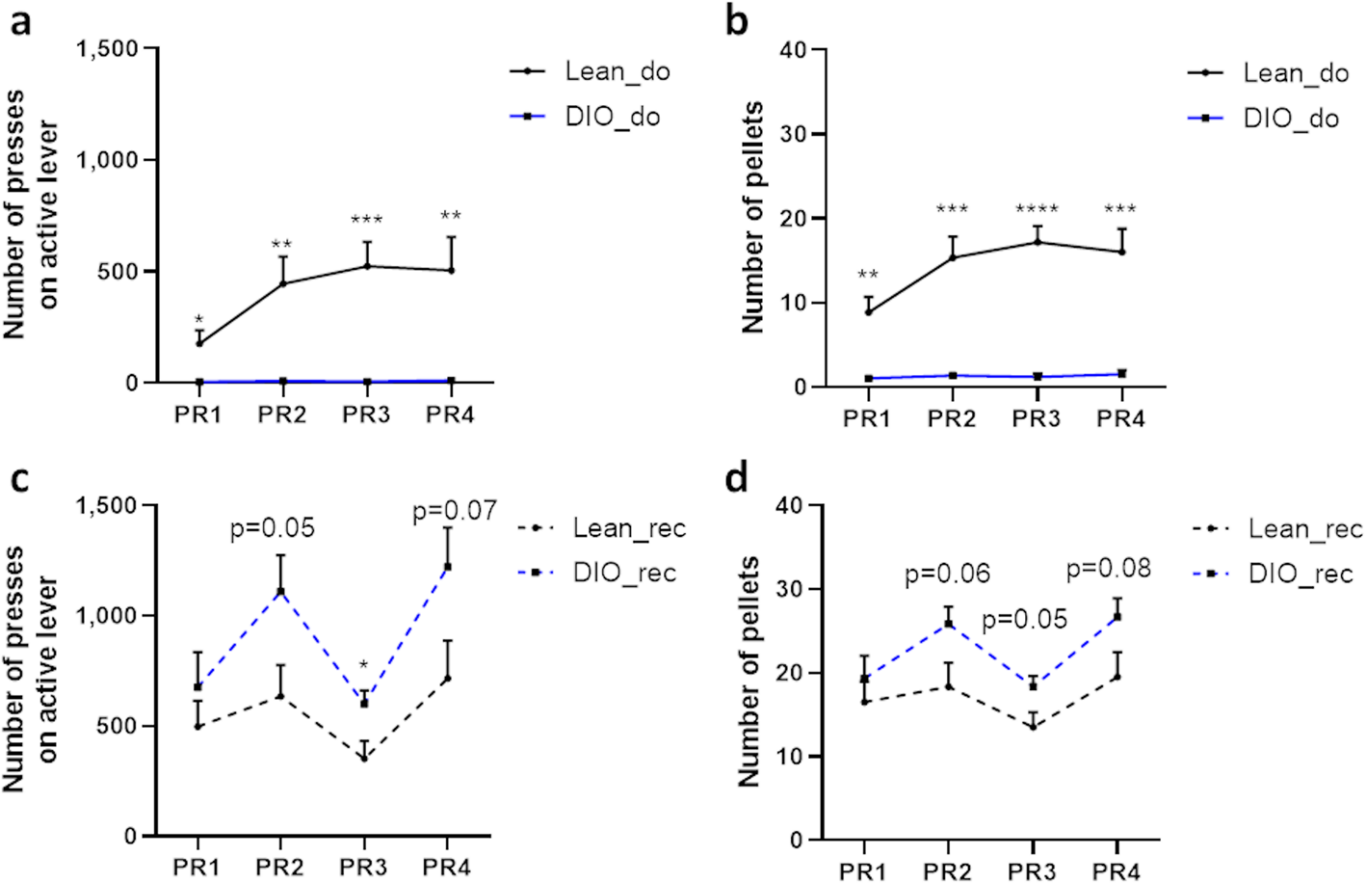
Gut microbes from obese donors lead to excessive motivation for food reward. Operant conditioning test showing **a** the number of active lever presses and **b** the number of pellets earned during the progressive ratio sessions (PR) by lean (Lean_do) and diet-induced obese donor mice (DIO_do). Operant conditioning test showing **c** the number of active lever presses and **d** the number of pellets earned during the progressive ratio sessions (PR) by gut microbiota recipient mice from lean (Lean_rec) and obese donors (DIO_rec). Data are shown as mean ± SEM (*n*=6/group). *p*-values were obtained after unpaired Student’s *t* test. *: *p*-value ≤ 0.05; ** : *p*-value ≤ 0.01; *** : *p*-value ≤ 0.001; **** : *p*-value ≤ 0.0001

To assess the wanting component or the motivation to obtain food reward, donor and recipient mice underwent an operant wall test in which they had to press on a lever to receive a rewarding sucrose pellet (Fig. 4). The first three sessions of the test were based on a fixed ratio (FR) principle: one food reward required one lever press. Then, in progressive ratios sessions (PR), mice had to press an incrementally increasing number of times (*n*+3) on the lever in order to obtain each new sucrose pellet, to assess their motivation to obtain a food reward.

Obese mice pressed significantly less on the lever during PR sessions as compared to lean mice (Fig. 4a). The number of reward pellets obtained is also significantly lower for obese than for lean donors during PR sessions (Fig. 4b). As PR sessions are the better reflection of the motivation to obtain a reward, our data show that obesity is associated with an alteration of the motivational component of the food reward. These data are consistent with results previously described in literature [26, 27].

Surprisingly, gut microbiota recipient mice from obese donors pressed more on the lever during PR sessions 2, 3, and 4 (*p*=0.05 during PR2, *p*<0.05 during PR3, *p*=0.07 during PR4), as compared to lean gut microbiota recipient mice (Fig. 4c). This tendency was reflected by the higher number of rewards obtained by the mice inoculated with gut microbes from obese donors during the PR sessions 2, 3, and 4 (Fig. 4d). These results suggest that gut microbiota recipient mice from obese donors behave in an opposite way as their obese donors in this test assessing the motivation to obtain a reward, since the former pressed approximately 100 times more on the lever to obtain a food reward than the latter. It is worth noting that the absolute values of number of lever presses are similar between Lean_rec and Lean_do groups (Fig. 4a, c). Gut microbiota recipient mice from obese donors showed particularly higher values of active lever presses (Fig. 4c), suggesting an excessive motivation for a food reward, rather than a normal motived behavior observed in lean conditions.

### Gut microbes from obese mice alter the brain reward system of recipient mice

**Fig. 5:**
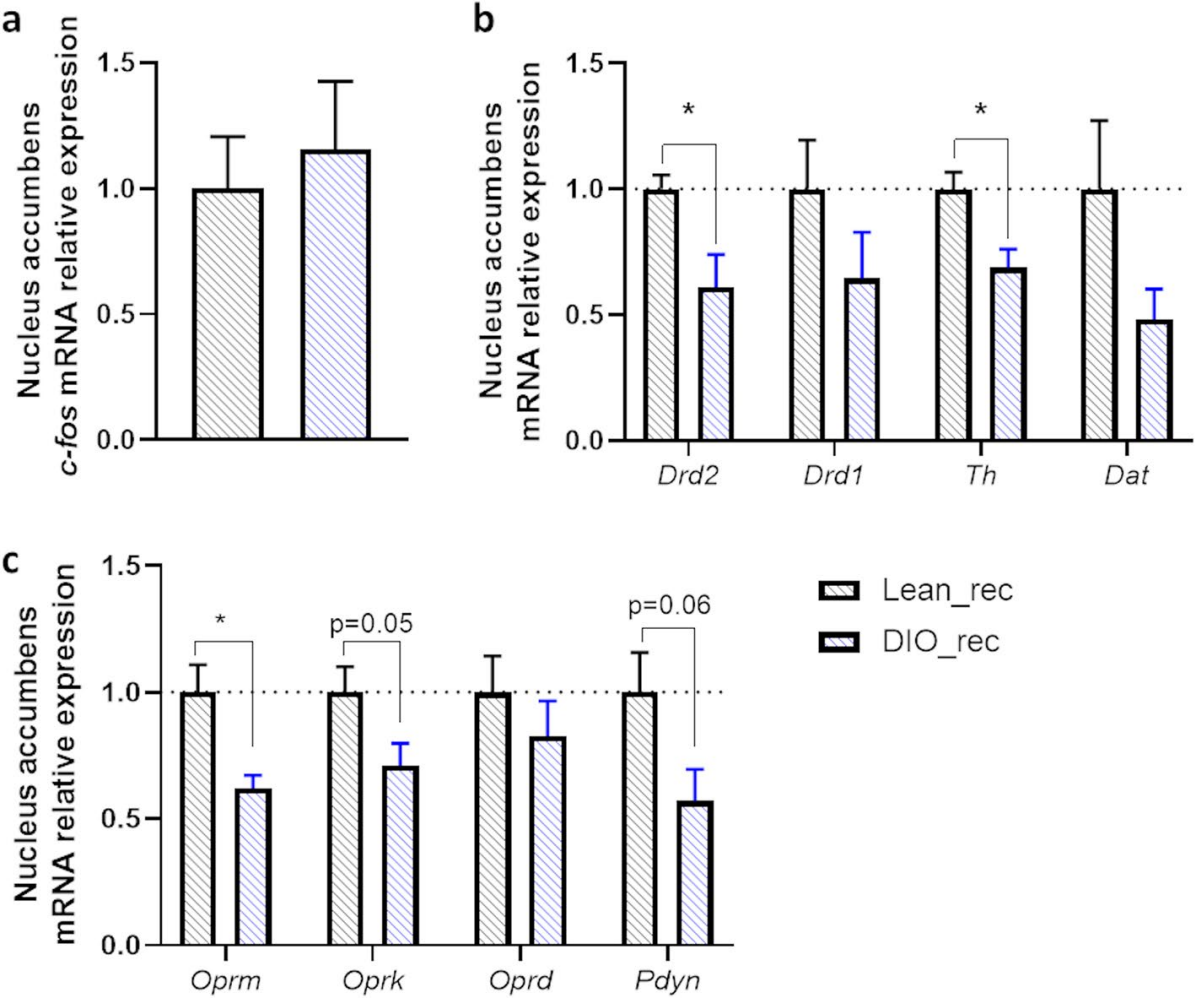
Dopaminergic and opioid systems of gut microbiota recipient mice from obese donors are downregulated. Nucleus accumbens mRNA expressions of **a** *c-fos*, **b** dopamine receptor 2 (*Drd2*), dopamine receptor 1 (*Drd1*), tyrosine hydroxylase (*Th*), dopamine transporter (*Dat*), and **c** µ-opioid receptor (*Oprm*), κ-opioid receptor (*Oprk*), δ-opioid receptor (*Oprd*), and pre-prodynorphin (*Pdyn*) measured by qPCR in gut microbiota recipient mice from lean (Lean_rec) and diet-induced obese donor mice (DIO_rec). Data are shown as mean ± SEM (*n*=7–8/group). *p*-values were obtained after unpaired Student’s *t* test or non-parametric Mann-Whitney test. * : *p*-value ≤ 0.05

To better understand the excessive motivation of mice recipient of gut microbes from obese mice for a food reward, we investigated the expression of neuronal activity, dopaminergic and opioid markers in the NAc of gut microbiota recipient mice from lean and obese donors (Fig. 5). We did not find any difference in the expression of *c-fos* mRNA, a marker of neuronal activity, in mice recipient of gut microbes from obese donors compared to mice recipient of gut microbes from lean donors (Fig. 5a). However, we found a significant decrease in the expression of dopamine receptor 2 (*Drd2*) and the enzyme synthetizing dopamine, tyrosine hydroxylase (*Th*), in the NAc of mice recipient of gut microbes from obese donors compared to mice recipient of gut microbes from lean donors. Dopamine receptor 1 (*Drd1*) and the dopamine transporter (*Dat*) tended to be reduced in mice transplanted with gut microbiota from obese mice compared to mice transplanted with gut microbiota from lean mice, but this did not reach significance (Fig. 5b).

Since the opioid system is also involved in food reward and has been shown to be blunted in obese conditions [5, 28], we measured the expression of some key markers and found that DIO_rec had a significant reduction in the NAc expressions of μ-opioid receptor (*Oprm*), a similar trend for reduction in κ-opioid receptor (*Oprk*, *p*=0.05) and the precursor of the dynorphin (*Pdyn*, pre-prodynorphin, *p*=0.06, Fig. 5c). The expression of δ-opioid receptor (*Oprd*) did not differ between Lean_rec and DIO_rec (Fig. 5c).

### Excessive motivation for food reward is not associated with modulations of homeostatic regulators of food intake

**Fig. 6:**
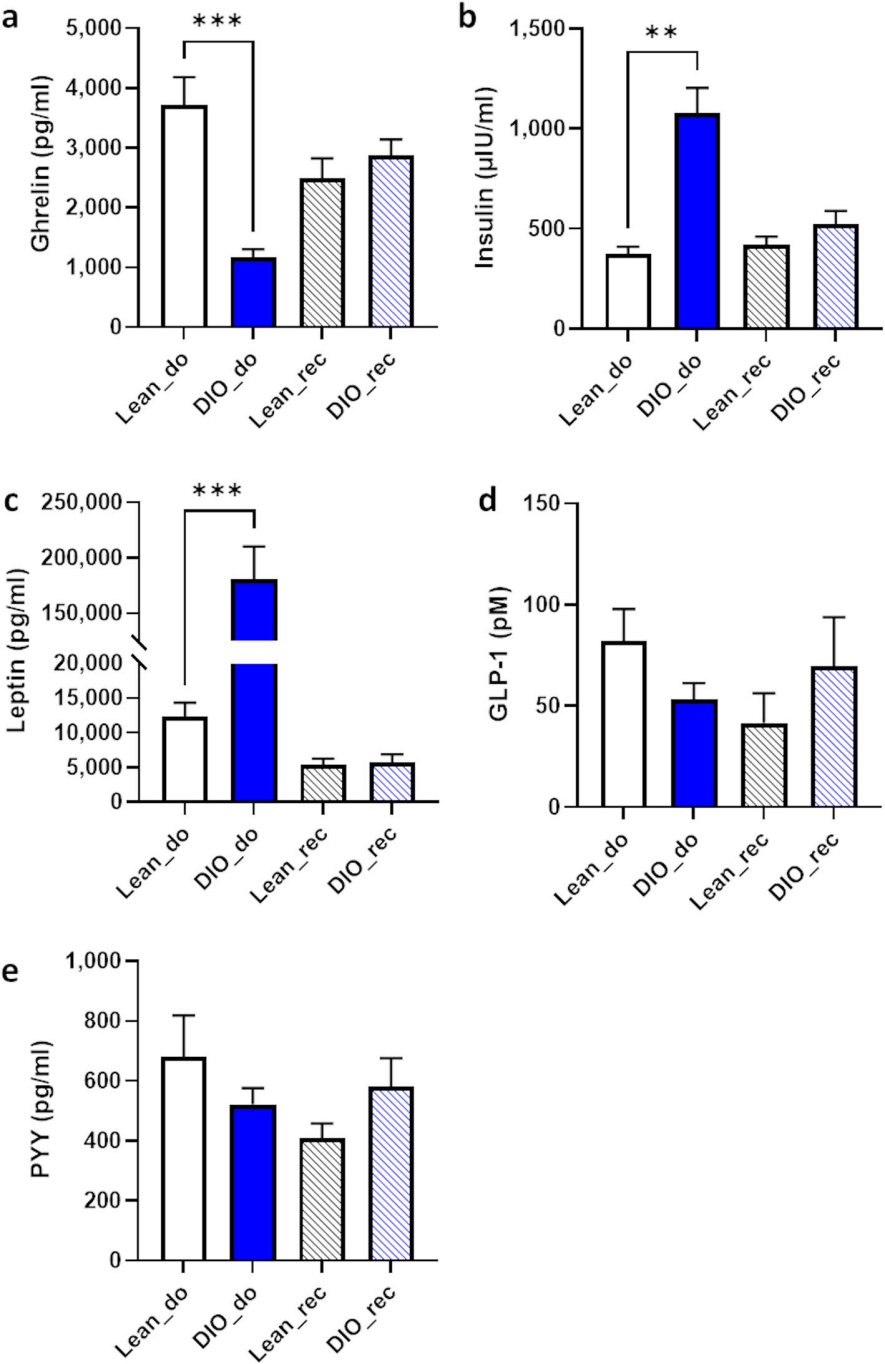
Homeostatic regulators of food intake are similar between recipient mice. Plasma concentrations of **a** ghrelin, **b** insulin, **c** leptin, **d** glucagon-like peptide-1 (GLP-1), and **e** peptide YY (PYY) in gut microbiota recipient mice from lean (Lean_rec) and diet-induced obese donor mice (DIO_rec). Data are shown as mean ± SEM (*n*=7–8/group). *p*-values were obtained after unpaired Student’s *t* test or non-parametric Mann-Whitney test between lean and obese (DIO) donor and recipient mice separately. ** : *p*-value ≤ 0.01; *** : *p*-value ≤ 0.001

To understand how gut microbes from obese mice could act on the behavioral and neuronal reward system in lean conditions (recipient mice), we analyzed several mediators of the gut-brain axis involved in the regulation of homeostatic food intake that are also able to influence the food reward system. Therefore, we measured ghrelin, insulin, leptin, total GLP-1 (active + inactive forms), and PYY in the plasma of recipient mice, as well as in donor mice. None of the homeostatic regulators analyzed in the plasma were different between gut microbiota recipient mice from lean and obese donors (Fig. 6). In contrast, we observed typical hormonal changes associated with obesity in the plasma of obese donor mice such as a significant decrease in ghrelin (Fig. 6a), a significant increase in insulinemia (Fig. 6b) and leptinemia (Fig. 6c) compared to lean mice. Plasma levels of GLP-1 and PYY were not significantly different between lean and obese donor mice (Fig. 6d, e).

### Excessive motivation for food reward is associated with changes in metabolomic profile

**Fig. 7:**
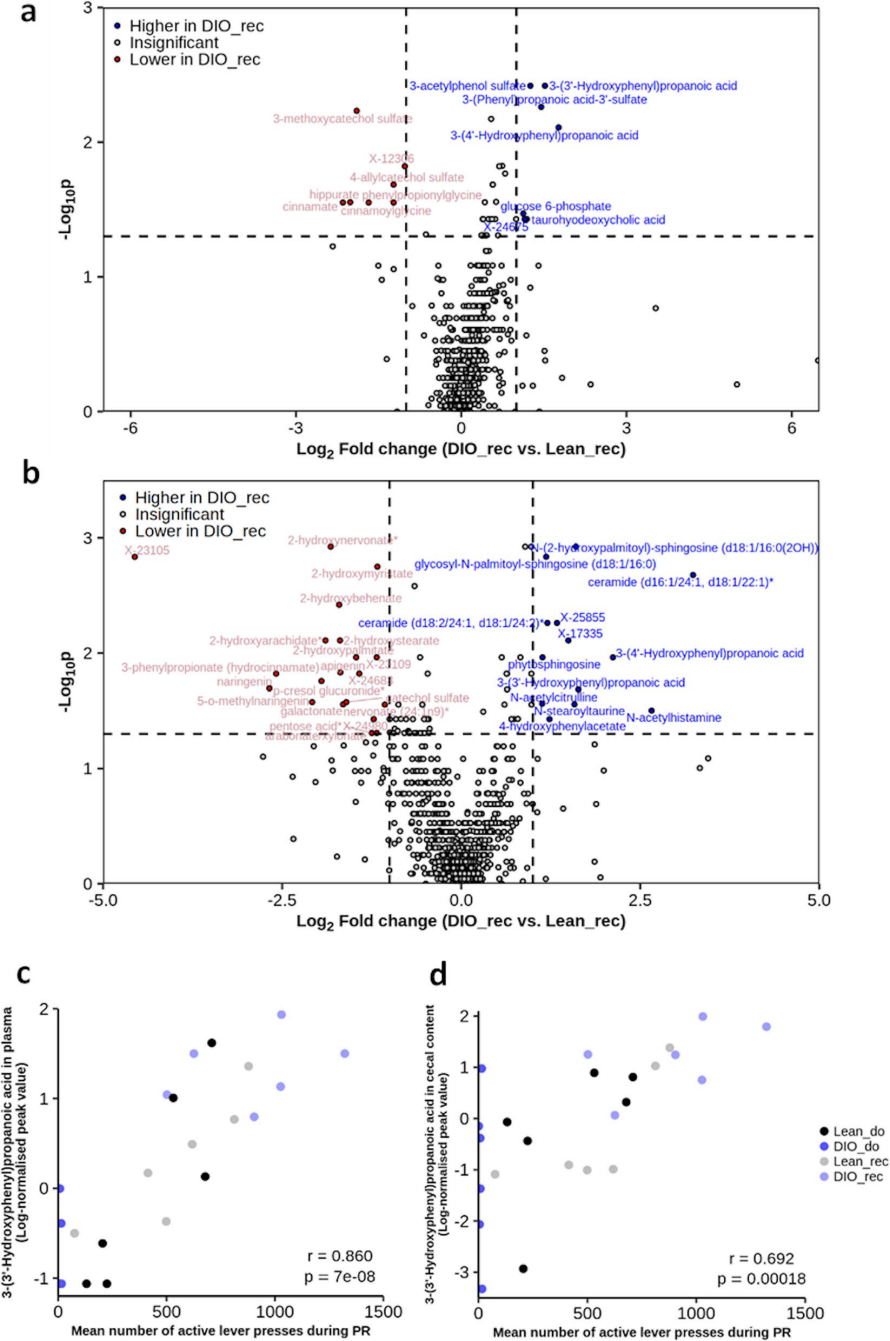
Untargeted metabolomics analysis reveals a metabolic candidate regulating the motivation for food reward. Volcano plots showing metabolites discriminant between gut microbiota recipient mice from lean and obese donors in **a** the plasma and **b** in the cecal content. Metabolites with labeled point were significantly down (red) or up (blue) in gut microbiota recipient mice from obese donors (DIO_rec) after non-parametric Wilcoxon test (*p*-value < 0.05) and were more than 2-fold changed compared to lean gut microbiota recipient mice (Lean_rec). Metabolites that did not satisfy the threshold of significance at Wilcoxon test and that were less than 2-fold changed compared to the lean gut microbiota recipient mice, appear in gray and are not labeled. The horizontal line corresponds to the *p*-value cutoff of 0.05 at non-parametric Wilcoxon test, the vertical lines correspond to the fold change cutoff of 2. See also Table 1 for the detailed list of the significantly different metabolites between gut microbiota recipient mice from lean and obese donors. Metabolites with a star (*) are partially characterized, and X metabolites are unidentified. Pearson’s correlations between 3-(3’-Hydroxyphenyl)propanoic acid concentrations in **c** the plasma or in **d** the cecal content of donor and gut microbiota recipient mice and the mean number of lever presses on the active lever during the progressive ratio (PR) sessions of the operant wall test

Since none of the hormonal mediators of the gut-brain axis that we analyzed were discriminant between gut microbiota recipient groups, we used untargeted metabolomics to identify potential metabolites involved in the regulation of the reward system and the motivational behavior. We screened both plasma and cecal samples of gut microbiota recipient mice from lean and obese donors. Univariate analyses such as volcano plots allowed us to illustrate metabolites affected (i.e., increased or decreased) by the gut microbiota from obese mice compared to gut microbiota from lean mice in the plasma and the cecal content of recipient mice (Fig. 7a, b, and Table 1). Applying a *p*-value set at 0.05 for the Wilcoxon test and considering a minimal 2-fold change, we identified 14 metabolites significantly modified in the plasma of DIO_rec group compared to Lean_rec (Fig. 7a and Table 1). In the cecal content, 33 metabolites were significantly different between Lean_rec and DIO_rec (Fig. 7b and Table 1). Two metabolites, namely 3-(3′-hydroxyphenyl)propanoic acid (33HPP) and 3-(4′-hydroxyphenyl)propanoic acid (34HPP), were similarly affected in the plasma and the cecum of mice transplanted with the gut microbiota from obese mice. Since these compounds are specific gut microbiota metabolites, they represented interesting candidate molecules possibly involved in mediating the effects of gut microbes on the food reward. Indeed, we speculated that they would be absorbed in the intestine and then exert an action on the reward system through the blood circulation. Interestingly, one study suggests that the 33HPP is able to cross the blood-brain barrier, probably through a carrier (like other phenolic compounds) [29].

**Table 1:** List of significantly different metabolites between gut microbiota recipient mice after untargeted metabolomics analysis

***Metabolites in plasma***	***Metabolites in cecal content***
**3-(3′- Hydroxyphenyl)propanoic acid**	2-Hydroxyarachidate*
3-(Phenyl)propanoic acid-3′-sulfate	2-Hydroxybehenate
**3-(4′- Hydroxyphenyl)propanoic acid**	2-Hydroxymyristate
3-Acetylphenol sulfate	2-Hydroxynervonate*
3-Methoxycatechol sulfate	2-Hydroxypalmitate
4-Allylcatechol sulfate	2-Hydroxystearate
Cinnamate	**3-(3′-Hydroxyphenyl)propanoic acid**
Cinnamoylglycine	**3-(4′-Hydroxyphenyl)propanoic acid**
Glucose 6-phosphate	3-phenylpropionate (hydrocinnamate)
Hippurate	4-Hydroxyphenylacetate
Phenylpropionylglycine	5-o-Methylnaringenin
Taurohyodeoxycholic acid	Apigenin
X-12306	Arabonate/xylonate
X-24675	Catechol sulfate
	Ceramide (d16:1/24:1, d18:1/22:1)*
	Ceramide (d18:2/24:1, d18:1/24:2)*
	Galactonate
	Glycosyl-N-palmitoyl-sphingosine (d18:1/16:0)
	N-(2-hydroxypalmitoyl)-sphingosine (d18:1/16:0(2OH))
	N-acetylcitrulline
	N-acetylhistamine
	N-stearoyltaurine
	Naringenin
	Nervonate (24:1n9)*
	p-cresol glucuronide*
	Pentose acid*
	Phytosphingosine
	X-17335
	X-23105
	X-23109
	X-24683
	X-24980
	X-25855

Metabolites significantly different between in gut microbiota recipient mice after non-parametric Wilcoxon test (*p*-value < 0.05) and more than 2-fold changed compared to lean gut microbiota recipient mice. Metabolites with a star (*) are partially characterized, and. X metabolites are unidentified. Metabolites in bold are common between the plasma and the cecal content

To further explore this hypothesis, and to make the link between metabolites in the plasma and the motivational behavior, we integrated all metabolites from the untargeted metabolomics analysis of donor and recipient mice into a correlation matrix with the mean active lever presses during PR sessions of the operant wall test as variable. After FDR correction, we discovered that the concentrations of 26 metabolites correlated significantly with the motivational parameter in both plasma and cecum (Additional file 1, Fig. 7c, d). The 33HPP appeared as the highest correlated metabolite in the plasma, potentially acting through the gut-brain axis (Fig. 7c).

These strong correlations suggest that this microbial metabolite could be a potential candidate involved in the effect of gut microbes on the motivational component of the food reward in gut microbiota recipient mice and acting via the blood circulation. Of note, we found reduced 33HPP in the plasma of obese mice compared to lean donor mice and in contrast, particularly increased 33HPP in the plasma and in the cecal content of mice under control diet, receiving a gut microbiota from obese donors (Additional file 2 a,b). In order to investigate if the 33HPP is able to act on the brain by passing through the blood-brain barrier, we measured in the prefrontal cortex (PFC) of the mice, the main conjugated metabolite of the 33HPP, the 3-(Phenyl)propanoic acid-3′-sulfate (33HPP-S) which represents the most abundant form of 33HPP. Interestingly, the quantification of the 33HPP-S in the PFC follows the same tendency as the quantification of 33HPP in the circulation, with an increase of the 33HPP-S in the PFC of mice transplanted with the gut microbiota from obese donors (Additional file 2c). Altogether, these results suggest that the 33HPP could reach the circulation and cross the blood-brain barrier to act on the brain.

As 33HPP is a gut-derived metabolite, we sought to identify bacteria producing 33HPP in the gut of recipient mice from obese donors. For this purpose, we correlated gut microbiota sequencing data from donor and recipient mice with the levels of 33HPP in the plasma and in the cecum (Additional file 3). We found that *Akkermansia* (Additional file 4a),* Muribaculum* (Additional file 4b) and, considering the scatterplots, especially *Prevotellaceae* (Additional file 4c), *Alloprevotella* (Additional file 4d) and *Parabacteroides* (Additional file 4e) were correlated with 33HPP levels in the plasma and in the cecal content.

### The gut microbiota-derived metabolite 33HPP influences the expression of key markers associated with reward in the NAc

Since gut microbiota recipient mice from obese donors showed excessive motivation and alterations in the expression of specific markers related to reward in the NAc (decrease of dopaminergic and opioid receptors), associated with a higher concentration of 33HPP in the cecum and the blood, we investigated whether 33HPP could have a direct impact on food reward in mice, independently of gut microbes. To this end, we administered 33HPP daily by intra-peritoneal injection to lean mice fed with a control diet. We evaluated the specific effect of 33HPP on dopaminergic and opioid receptors in the NAc and the impact on the operant conditioning.

**Fig. 8:**
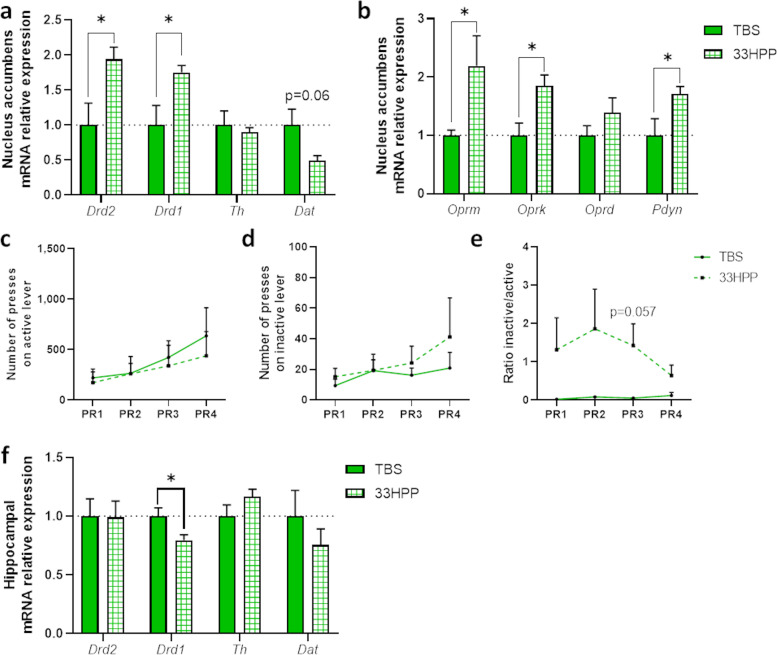
33HPP modulates the food reward system and alters the memory.** a** Nucleus accumbens mRNA expressions of dopamine receptor 2 (*Drd2*), dopamine receptor 1 (*Drd1*), tyrosine hydroxylase (*Th*), dopamine transporter (*Dat*), **b** µ-opioid receptor (*Oprm*), κ-opioid receptor (*Oprk*), δ-opioid receptor (*Oprd*), and pre-prodynorphin (*Pdyn*) measured by qPCR in mice injected with TBS (TBS) and with 3-(3’-Hydroxyphenyl)propanoic acid (33HPP). Operant conditioning test showing **c** the number of active lever presses, **d** the number of inactive lever presses, and **e** the ratio between the number of inactive lever presses on the number of active lever presses during the progressive ratio sessions (PR) by mice injected with TBS (TBS) and mice injected with 33HPP (33HPP). **f** Hippocampal mRNA expressions of *Drd2*, *Drd1*, *Th*, and *Dat* in mice injected with TBS (TBS) and mice injected with 33HPP (33HPP). Data are shown as mean ± SEM (*n*=6/group). *p*-values were obtained after unpaired Student’s *t* test or non-parametric Mann-Whitney test

Surprisingly, we found an increased expression of *Drd2* and *Drd1* in lean mice injected with 33HPP compared to lean mice injected with the vehicle (Fig. 8a). The expression of *Dat* tended to be decreased after 33HPP supplementation (*p*=0.06, Fig. 8a), whereas the expression of the *Th* was not significantly affected (Fig. 8a). In the same line, the expressions of *Oprm* and *Oprk* were increased in 33HPP-treated mice as compared to TBS-treated mice, as well as the expression of *Pdyn* (Fig. 8b.) The expression of the *Oprd* was not significantly affected by 33HPP administration (Fig. 8b.).

However, 33HPP did not influence the motivation of lean mice to obtain a food reward since the number of presses on the active lever (leading to the delivery of a food reward) was equivalent to that of mice injected with the vehicle (Fig. 8c). Interestingly, the number of presses on the inactive lever (not leading to the delivery of a food reward) was slightly higher in 33HPP mice compared to TBS mice (Fig. 8d). This trend was even more apparent when calculating the ratio between inactive and active lever presses (Fig. 8e). Mice injected with 33HPP were unable to distinguish the active from the inactive lever.

Since these results suggest a dysfunction in memory and learning process, we investigated dopaminergic markers in the hippocampus, a brain area also implicated in the food reward system as the main regulator for memory and learning process. Lean mice receiving 33HPP had significantly lower expression of *Drd1* compared to placebo-treated mice, whereas the other dopaminergic markers analyzed were not changed (*Drd2, Th, Dat*) (Fig. 8f). This suggests that 33HPP is inducing an alteration of the memory and learning process associated with the reward system.

## Discussion

In a previous proof-of-concept study using FMT from obese mice donors, we demonstrated that the gut microbiota played a causal role in the dysregulations of the liking component of the food reward system observed during obesity. In this study, we confirmed the concept and we thus aimed to decipher the role of the gut microbiota in obese conditions in two other aspects of the reward system, i.e., the learning and motivational components, by using the CPP and the operant conditioning tests respectively. These behavioral approaches were applied in both lean and obese mice, as well as in lean recipient mice transplanted with gut microbiota from lean or obese donor mice. Interestingly, we discovered that gut microbes from obese mice affect the brain reward system of lean acceptor mice by inducing excessive motivation for food reward. Using untargeted metabolomics analysis on samples from gut microbiota recipient mice, we aimed to identify new metabolites potentially contributing to the altered food reward system during obesity and to the excessive impulse to obtain a food reward observed after inoculation with an obesity-associated gut microbiota. We then administered a newly identified metabolic candidate (33HPP) in lean mice and demonstrated that this metabolite could be implicated in the gut-brain axis, modulating the brain reward system and affecting behavior.

Since we aim to identify the specific roles of gut microbes on the reward system, the fecal transplantation model appears to be the more appropriate approach. In this study, the FMT from either lean or obese donor into lean recipient mice was effective to transfer bacterial genera from donors into recipient mice. Indeed, we have shown that a treatment with broad-spectrum, poorly absorbed, antibiotics and a laxative prior to the FMT was efficient to deplete more than 99% of the cecal bacterial load of recipient mice [22]. Moreover, the use of young mice (3-week-old) for the FMT further improved the engraftment of donor gut microbes [30]. The high percentage of similar genera found in our study between donors and recipients confirms the efficient engraftment of gut microbes from donors into the recipient mice. Our FMT protocol also allows to cluster the gut microbiota compositions of donor and recipient mice as illustrated by the PCoA, based on the presence or absence of some bacteria (Jaccard distance). In this study, transfer of the gut microbiota from obese mice did not replicate the obese phenotype from HFD-induced obese donor into recipient mice (i.e., increased body weight gain and fat mass development). Since mice recipient of gut microbes from obese mice were maintained under control, low-fat diet during the whole protocol, and it has been shown that the type of diet predominates to influence the phenotype rather than the FMT itself, this could explain our observations [31].

When investigating the reward system from a behavioral point of view, the training sessions with Reese’s^®^ were efficient to induce a conditioned behavior in donor and recipient mice, confirming the validity of our food-induced CPP protocol. Lean and obese donor mice reverse their initial preference for a particular test chamber. Interestingly, unlike lean donors, obese donors displayed an altered learning component of the food reward since they tend to spend less time in the palatable-associated compartment. Such cognitive impairments observed with CPP test and associated with obesity have already been described in the literature [32–34]. In gut microbiota recipient mice, both groups seemed able to associate one side of the cage with food-induced pleasure since they both spent more time in the palatable food-associated chamber during the test as compared to the pre-test. However, only mice transplanted with a gut microbiota from lean mice were able to reverse their original preference after training sessions. Our data demonstrate that the altered learning component of the food reward associated with obesity was partially transferred through FMT.

We further characterized the reward behavior of obese mice and we found a reduced motivation for food reward, as already described in the literature [27, 32]. The altered learning component of the food reward and the decreased motivation in obese mice that we observed are a good reflection of their hypofunctional brain reward system [10, 35, 37]. Surprisingly, mice transplanted with fecal material from obese donors did not behave during the operant wall test like their obese donors. On the contrary, they pressed more often, and even excessively on the lever to obtain sucrose pellets. This excessive motivation could induce an over-activation of the reward system, which afterwards downregulates as a negative feedback. This hypothesis could explain the downregulation of key mediators of dopamine and endorphin signaling (*Drd2*, *Th* and *Oprm*, *Oprk*, *Pdyn* respectively) that we observed in mice transplanted with a gut microbiota from obese donors [36]. The exaggerated motivation of mice transplanted from obese donors associated with decreased *Drd2* expression could reflect the compulsive behavior that is described in the context of obesity. In 2010, Johnson and Kenny demonstrated that obese rats with decreased expression of *Drd2* showed an addiction-like behavior to eat palatable food [37]. Moreover, it has already been shown that mice fed with a HFD for 6 weeks then suddenly switched on a low-fat diet showed increased operant response for sucrose and fat reward pellets, suggesting craving for palatable food [38]. This was associated with modifications in dopaminergic transmission in the Nac including a decrease in the expression of *Th*, as we also observed in gut microbiota recipient mice from obese donors fed with a low-fat diet and pressing excessively on the lever to obtain sucrose rewards [38].

Food anticipatory activity induced by food cues (after conditional stimulus) and palatable food ingestion induce an increase in neuronal activity in reward-related area in the brain, including in the NAc, as reported by the increase in c-Fos immunoreactivity [39]. Therefore, we investigated the expression of *c-fos* mRNA in the NAc of mice transplanted with gut microbiota from lean and obese donor mice to evaluate if a difference in neuronal activity can be observed. We did not observe any difference in *c-fos* mRNA expression between Lean_rec and DIO_rec mice. Additional experiments including immunohistology of the brain could be interesting to perform in order to deeper investigate differences in specific neuron activity associated with obese gut microbiota compared to lean gut microbiota.

Homeostatic regulators and their effects on behavioral and neuronal food reward system are well described in the literature [15, 40, 41]. It is well established that food restriction leads to increased reward response to palatable stimulus and that this is facilitated by the decrease in plasma leptin and insulin [42]. In fact, injections of insulin or leptin, reverse their respective effects and decrease the response in CPP and the operant wall tests [23, 43, 44]. Importantly, mice under a moderate HFD, with similar body weights and leptinemia as a control group, showed increased PR response during the operant wall test [45]. This demonstrates that body weight gain and adipose signals are major drivers to the decreased operant response to sucrose pellet under HFD. In our study, gut microbiota recipient mice from obese donors do not show any leptin or insulin elevation typical from obesity. This suggests that their behavior and changes in their brain reward system are mainly due to obese gut microbes rather than homeostatic mediators controlling the reward responses. These results assessing the learning and motivational components of the food reward complete our previous data showing that the liking component was transferable through FMT [22]. Since the liking, the wanting and the learning components of the food reward involve distinct brain areas, mediators and signaling, it could explain why we observe different results for these three components [6, 7].

Importantly, sweet taste is also a strong contributor to the reward process. Indeed, the taste perception at the level of the mouth, and the post-ingestive signals at the level of the gut, both contribute to the increase in the reward response and dopamine release in the brain [46]. That said, we focus on the gut-brain axis to assess the reward response modulated by gut microbes.

In the second part of our study, we identified 33HPP as a metabolite being particularly increased in gut microbiota recipient mice from obese donors and correlating with the mean active lever presses during the operant conditioning. This metabolite is produced exclusively by bacteria (and not by the host) including by the genera *Clostridium*, *Eubacterium*, and *Escherichia* and could be a degradation product from several polyphenols (chlorogenic acid, quercetin, naringenin, apigenin…) [47–52]. Even if the source of 33HPP in our study remains unclear, some polyphenol compounds (naringenin, apigenin) appeared to be present in the plasma and in the cecal content of recipient mice after metabolomics analyses and could then represent a potential source of 33HPP. 34HPP, another potentially interesting metabolite that we identified in this context, can be produced through the degradation of DOPA by the gut microbiota, and some bacteria including *Enterococcus faecalis* and *Enterococcus faecium* can produce DOPA [53, 54]. This may represent a source of 34HPP in the cecum and in the blood of recipient mice. In the literature, one study showed that administration of polyphenols increases 33HPP levels in the blood and in the brain suggesting the capacity of 33HPP to pass the blood-brain barrier [29]. Moreover, our data support this hypothesis since we were able to quantify the 33HPP-S in the PFC of the mice with an increase of 33HPP-S in the PFC of mice transplanted with gut microbiota from obese mice. Even if they have not been described so far as producers of 33HPP, unknown *Prevotellaceae*, *Alloprevotella*, and *Parabacteroides* also correlated with 33HPP levels in the blood and in the cecal content. This strengthened our previous findings that proposed a role for *Parabacteroides* in the regulation of the food reward system [22].

To causally investigate the effects of 33HPP on the food reward, we injected 33HPP to lean mice under control diet. We found that 33HPP itself had no significant effect on the motivational component of the food reward (number of lever presses) despite an increase in the expression of dopaminergic and opioid markers. However, the learning process seemed altered in mice injected with 33HPP compared to placebo-treated mice. In fact, 33HPP-treated mice were apparently not able to develop goal-directed behavior as they did not make the distinction between the active lever delivering a sucrose pellet and the inactive lever. Moreover, dopaminergic marker *Drd1* was decreased in the hippocampus of mice injected with 33HPP, suggesting an alteration of their reward memory and learning process. Interestingly, some studies showed that phenolic acid compounds, among which 33HPP, were elevated in children with autism spectrum disorder (ASD) [55–57]. Among a panel of behavioral symptoms, ASD is characterized by stereotypical actions, i.e., repetitive, invariant behavior pattern with no obvious goal or function. This pattern could be compared to lever pressing without aiming for the food reward. In the context of ASD, a recent study has shown that an oral drug adsorbing small aromatic or phenolic molecules, AB-2004, was associated with decreased levels of phenolic acids including 34HPP in the blood of mice [58]. Treatment with AB-2004 significantly improves compulsivity assessed with the marble burying test. This study confirms the alterations in the behavior such as anxiety or compulsivity driven by gut microbial production of phenolic compounds and suggests a promising therapeutic approach through the adsorption of phenolic compounds [58].

We discover here that 33HPP stimulates the expression of key dopaminergic and opioid markers involved in the reward system (*Drd2*, *Drd1*, *Oprm*, *Oprk*, *Pdyn*) in control-fed mice. By combining the results from the 33HPP experiment and the FMT experiment, we speculate that the elevated 33HPP in the blood and in the cecum of mice receiving a gut microbiota from obese donors made them particularly sensitive to a food reward, 33HPP being the main driver for the over-expression of dopaminergic and opioid markers.

In conclusion, we confirmed that obese mice have a dysregulation of the learning and the wanting components of the food reward. We demonstrated that gut microbes play a role in the regulation of food reward and could be responsible for compulsive behavior and excessive motivation to obtain sucrose pellets. Moreover, obese gut microbes affected dopaminergic and opioid markers involved in reward system. We identified 33HPP as particularly increased in mice recipient of gut microbes from obese mice, and we were able to demonstrate its effects as key mediator of the gut-brain axis controlling the reward response to palatable food.

## Methods

### Mice and experimental design

All mouse experiments were approved by the ethical committee for animal care of the Health Sector of the UCLouvain, Université catholique de Louvain under the specific numbers 2017/UCL/MD/005 and 2021/UCL/MD/060 and performed in accordance with the guidelines of the local ethics committee and in accordance with the Belgian Law of May 29, 2013, regarding the protection of laboratory animals (agreement number LA1230314).

### Donor mice

A cohort of 8-week-old SOPF male C57BL/6J mice (15 mice, *n*=7–8 per group) (Janvier laboratories, France) were housed in a controlled environment (room temperature of 22 ± 2 °C, 12h daylight cycle) in groups of two mice per cage, with free access to sterile (irradiated) food and sterile (autoclaved) water. Upon delivery, mice underwent an acclimatization period of 1 week, during which they were fed a control diet (CT, AIN93Mi, Research Diet, New Brunswick, NJ, USA). Then, mice were randomly divided into two groups and were fed for 14 weeks with control low-fat diet (CT, AIN93Mi) or a HFD (60% fat and 20% carbohydrates (kcal/100g) D12492i, Research diet, New Brunswick, NJ, USA). Body weight was recorded once a week. Body composition was assessed weekly by using 7.5 MHz time domain-nuclear magnetic resonance (TD-NMR, LF50 Minispec, Bruker, Rheinstetten, Germany). After 11 weeks of follow-up, the mice were transferred to behavioral cages to perform the conditioned place preference test and the operant wall test. During these tests, mice were food-restricted and body weight was maintained at 85% of the initial body weight (before the behavioral tests). The caloric restriction allowed to potentiate the reward response to the stimulus [23, 24].

### Recipient mice

A cohort of 3-week-old SOPF male C57BL/6J mice (15 mice, *n*=7–8 per group) (Janvier laboratories, France) were housed in a controlled environment (room temperature of 22 ± 2 °C, 12h daylight cycle) in groups of two mice per cage, with free access to sterile (irradiated) food and sterile (autoclaved) water. Mice were fed a low-fat control diet (CT, AIN93Mi) during the entire procedure (before, during, and after gut microbiota transplantation). Body weight was recorded once a week. Body composition was assessed weekly by using 7.5 MHz time domain-nuclear magnetic resonance (TD-NMR, LF50 Minispec, Bruker, Rheinstetten, Germany). After 5 weeks of follow-up, the mice were transferred to behavioral cages to perform the conditioned place preference test and the operant wall test. During these tests, mice were food-restricted and body weight was maintained at 85% of the initial body weight (before the behavioral tests). The caloric restriction allowed to potentiate the reward response to the stimulus [23, 24].

### Fecal microbiota transplantation

During the experiment with donor mice, fecal samples were collected in sterile containers and immediately diluted (1:10 w/vol) in sterile PBS added with a mixture of antioxidants (0.5% ascorbic acid 0.5% L-cystein, 0.5% glutathione) and cryoprotectants (5% trehalose, 5% sorbitol). This suspension was filtered using 100-µm filters. Then the filtrate was aliquoted in anaerobic conditions before storage at −80°C. Three CT-fed mice and four HFD-fed mice from the donor cohort were selected as fecal microbiota donors for seven or eight recipient mice per group with 1 donor for 1, 2, or 3 recipient mice, according to quantity of feces from donors. As previously described, prior to gut microbiota inoculation, 3-week-old SOPF recipient mice were depleted in intestinal microbiota by daily gavage of a broad-spectrum, poorly absorbed mix of antibiotics during 5 days (100 mg/kg of ampicillin, neomycin, and metronidazole and 50 mg/kg of vancomycin diluted in sterile water) added with antifungal (amphotericin B 1mg/kg)[30, 59]. Antibiotic treatment was then followed by a bowel cleansing with the administration of 800µl of PEG solution (PEG/Macrogol 4000, Colofort, Ipsen, France) by oral gavage in two times at 30-min intervals after a 2-h fasting. Colonization was then achieved by intragastric gavage with 200 µl of inoculum three times a week for 1 week, then once a week until the end of the experiment. During inoculation, mice were transferred into clean cages 3 times a week. All recipient mice were kept under CT diet (CT, AIN93Mi).

### 33HPP experiment

A cohort of 7-week-old SOPF male C57BL/6J mice (20 mice, *n*=10 per group) (Janvier laboratories, France) were housed in a controlled environment (room temperature of 22 ± 2 °C,12h daylight cycle) in groups of two mice per cage, with free access to control sterile (irradiated) food (CT, AIN93Mi) and sterile water. Upon delivery, mice underwent an acclimatization period of 1 week. Then, mice were randomly divided in two groups and were injected daily with 100µl of a vehicle solution (TBS) or a solution containing 33HPP (25mg/kg) during 4 weeks. After 2 weeks of follow-up, the mice were placed in behavioral cages to perform the operant wall test. During this test, mice were food-restricted and body weight was maintained at 85% of the initial body weight (before the behavioral tests). The caloric restriction allowed to potentiate the reward response to the stimulus [23, 24].

### Food preference test

During 3 h in the daylight, mice were exposed to two diets: a low-fat, control diet (CT, AIN93Mi, Research diet, New Brunswick, NJ, USA) or a high-fat high-sugar diet (HFHS, 45% fat and 27.8% sucrose (kcal/100 g) D17110301i, Research diet, New Brunswick, NJ, USA) in metabolic chambers (Labmaster/Phenomaster, TSE systems, Germany) at 3 different days. Sensors recorded the precise food intake of each diet every 15 min. The food intakes were recorded during a 3-h session during the light phase, in satiated state (access to food ad libitum before and after the test).

### Conditioned place preference test

The learning component of the food reward is evaluated in donor and recipient mice by a CPP performed in the end of the light phase on a biased apparatus (Phenotyper chambers, Noldus, The Netherlands) as previously described with some modifications [60]. The behavioral cage is separated in two compartments characterized with smooth or rough floor and black or striped walls. All the compartments were completely cleaned before and after each session. Each session (pre-test, trainings, test) lasts exactly 30 min. Locomotor activity is recorded with infrared camera monitoring system and analyzed with the provided software (EthoVision XT 14). On day 1, a pre-test is used to determine the less preferred compartment in baseline (the one in which the mouse spent spontaneously less time) and is defined as the reward-associated compartment (biased CPP method). From day 2 to day 9, donor and recipient mice underwent eight trainings with or without a rewarding stimulus (Reese’s^®^), in the less and in the most preferred compartment respectively (4 sessions in each compartment). During the test, the mouse is free to run in each compartment of the cage (in absence of rewarding stimulus), and the time spent in each compartment is recorded (analyzed with the provided software (EthoVision XT 14)). Preference score is based on the difference of time spent (s) in the palatable food-associated side vs the time spent in the neutral-associated side of the cage during the pre-test and the test.

### Operant wall test

The wanting component is linked to the motivation to obtain a reward and is evaluated by an operant wall test in donor and recipient mice as previously described with some modifications [27, 60]. Each session of the test was conducted during the end of the light phase, in operant conditioning chambers (Phenotyper chambers, Noldus, The Netherlands), and analyzed by the provided software (Ethovision XT 14). Briefly, the mice had intermittent access to an operant wall in their home cages. The operant wall system is composed of two levers and two lights and a pellet dispenser. One lever is arbitrarily designated as active, meaning that pressing on this lever initiates the delivery of a sucrose pellet (5-TUT peanut butter flavored sucrose pellet, TestDiet, St. Louis, MO) and is associated with a light on. On the other side, another lever associated with a light off is arbitrarily designated as inactive and will never deliver a reward. Mice were trained for the system twice overnight on a FR schedule (one lever press corresponds to one reward), then underwent 4 sessions of 1h30. Mice were then shifted to PR sessions (2h), the number of lever press to obtain a reward is incrementally increased (*n*+3) for every pellet.

### Tissue sampling

At the end of each experiment, mice were maintained under caloric restriction before anesthesia with isoflurane (Forene, Abbott, England). This aims to mimic the conditions of the behavioral tests. Then, the mice were euthanized by exsanguination and cervical dislocation. Blood was sampled from the portal and cava veins. Nac and PFC was precisely dissected, the cecal content was harvested and immediately immersed into liquid nitrogen, then stored at −80°C for further analysis.

### RNA preparation and quantitative PCR analysis

Total RNA was extracted from the Nac and the hippocampus using TriPure reagent (Roche). Complementary DNA was prepared by reverse transcription of 1µg total RNA using the GoScript Reverse Transcriptase kit (Promega, Madison, WI, USA). Real-time PCR was performed with the QuantStudio 3 real-time PCR system (Thermo Fisher, Waltham, MA, USA). *Rpl19* RNA was chosen as the housekeeping gene. All samples were performed in duplicate, and data were analyzed according to the 2^−ΔΔCT^ method. The identity and purity of the amplified product were assessed by melting curve analysis at the end of amplification. Sequences of the primers used for real-time PCR are available in Additional file 5.

### DNA isolation from mouse cecal samples, total bacteria quantification, and 16S amplicon sequencing

Cecal contents were collected and kept frozen at −80 °C until use. Metagenomic DNA was extracted from the cecal content using a QIAamp DNA Stool Mini Kit (Qiagen, Hilden, Germany) according to the manufacturer’s instructions with modifications [61]. The bacterial load in the cecal content was quantified using qPCR as described above, with universal bacterial primers (Additional file 5). The V1–V3 region of the 16S rRNA gene was amplified from the cecal microbiota of the mice using the following universal eubacterial primers: 27Fmod (5′-AGRGTTTGATCMTGGCTCAG-3′) and 519Rmodbio (5′-GTNTTACNGCGGCKGCTG-3′). Purified amplicons were sequenced using an Illumina MiSeq following the manufacturer’s guidelines. Sequencing was performed at MR DNA (www.mrdnalab.com, Shallowater, TX, USA). Sequence reads were demultiplexed and processed using the QIIME2 pipeline (q2cli 2020.11.1) [62], including primer removal using cutadapt [63] and denoising with DADA2 [64] using the following options in DADA2: maximum expected error = 2, truncation length = 280 nt. To ensure quality, only forward sequencing reads were used. For the 30 samples analyzed, 971 ASVs have been identified. These were decontaminated by mapping to the mouse genome GRCm39 (GCF_000001635.27) and, after taxonomic assignment, by aligning unassigned ASVs to the non-redundant nucleotide database of the National Center for Biotechnology Information using BLAST [65], resulting in 958 decontaminated ASVs. The taxonomic assignment of ASVs was performed using a classifier based on the SILVA 138.1 SSURef NR99 database [66] that was dereplicated and trimmed to the V1–V3 region using RESCRIPt. After this processing, samples contained between 21,056 and 37,447 sequence reads, with the median and mean number of sequence reads of 29,687 and 29,504, respectively. Diversity metrics were calculated using a sampling depth of 21,056 reads, and PCoA was performed using the Jaccard distances and unweighted UniFrac distances with QIIME2. The PCoA plots were visualized using the “tidyverse” collection of R packages “tidyverse”. The sequencing data were submitted to the European Nucleotide Archive (ENA/EBI).

### Plasma multiplex analysis

Plasma levels of total GLP-1, PYY, total ghrelin, insulin, and leptin were measured by multiplex assay kits based on chemiluminescence detection and following the manufacturer’s instructions (Meso Scale Discovery, Gaithersburg, MD). Analyses were done using a QuickPlex SQ 120 instrument (MSD) and DISCOVERY WORKBENCH®4.0 software.

### Untargeted metabolomics analysis

Cecal and plasma samples were used for untargeted metabolomics analyses by UPLC-MS/MS (Metabolon, USA). To identify potential metabolites involved in the gut-to-brain axis and contributing to the reward system, the metabolomic data were then analyzed using R and R packages “stats” and “tidyverse”. For each compound, the raw peak areas were median normalized and the minimum value was imputed for the missing values, followed by a log-transformation. Metabolites with both a fold-change > 2 and a *p*-value < 0.05 after the Kruskal-Wallis test were considered significant and the results were visualized with a volcano plot. Raw data obtained from untargeted metabolomic analysis in the cecal content and plasma of mice are available in Additional file 6 and 7, respectively.

### 33HPP-S quantification

33HPP-S was extracted from PFC as described by Angelino et al., with 20 mg of PFC and 2 × 90 µL of acetonitrile acidified with 2% formic acid [67]. Quantification of 33HPP-S was performed by ultraperformance liquid chromatography coupled with quadrupole – time of flight (UPLC-QToF), using an Acquity I-Class UPLC coupled with a Synapt G2-Si QToF (Waters, Milford, MA, USA). Five microliters of PFC extract was injected onto an ACQUITY Premier HSS T3 column (2.1 × 100 mm, 1.8 µm) protected with a ACQUITY Premier HSS T3 VanGuard FIT pre-column (2.1 × 5 mm, 1.8 µm) kept at 40 °C. Chromatographic separation was achieved at 0.4 mL/min isocratically with 98% mobile phase A (0.01% formic acid in water) for 0.4 min, followed by a linear gradient to 45% mobile phase B (0.01% formic acid in acetonitrile) over 6.35 min. Then, column was washed-out for 4.45 min with 95% mobile phase B and re-equilibrated with initial conditions for 3.6 min. MS data were acquired as described elsewhere with slight modifications [68]. Data were acquired in sensitivity mode (resolution ≈ 15,000), and the source temperature, desolvation temperature, desolvation gas, and capillary voltage were set, respectively, to 150 °C, 500 °C, 1000 L/h, and −0.8 kV. 33HPP-S was identified as reported by Lessard-Lord et al. and was quantified as 3-(4′-hydroxyphenyl)propanoic acid-3′-sulfate (Toronto Research Chemicals, Toronto, Canada) equivalent [68].

### Statistical analysis

Statistical analyses were performed using GraphPad Prism version 9.1.2 for Windows (GraphPad Software, San Diego, CA, USA) except for microbiota analyses as described above. Data are expressed as mean ± SEM. Differences between two groups were assessed using two-tailed unpaired Student’s *t* test. In case variance differed significantly between groups according to the Fisher test, a non-parametric (Mann-Whitney) test was performed. Two-way ANOVA was used if repeated measurements, followed by Bonferroni post hoc test. Differences in microbial beta-diversity were assessed in QIIME2. Correlation of metabolite concentrations, either in plasma or in cecal content, and the wanting component of the reward system was performed using Pearson correlation on the log-transformed, imputed peak values from the metabolomic analysis, and the mean number of lever presses on the active lever during the PR sessions of the operant wall test. The metabolites that significantly correlated (FDR-adjusted *p*-value < 0.05) with the active lever presses in both plasma and cecal content were considered as relevant. Similarly, correlation of metabolite concentrations, either in plasma or in cecal content, and microbial genera in the cecal content was performed using the Spearman correlation on the log-transformed, imputed peak values from the metabolomic analysis, and the genus abundances obtained by scaling the genus-level relative frequencies with the qPCR-based total bacterial abundances in the cecum. To ensure the relevance of correlations, only genera that appeared in at least 10 samples were considered. Two samples that were outliers for total bacterial abundances were omitted from this analysis. Correlations were visually inspected using scatterplots (Additional file 4).

## Supplementary Information

**Additional file 1. **Metabolites correlating significantly in the plasma and in the cecum with the mean active lever presses after Pearson’s correlation and False Discovery Rate. (FDR).

**Additional file 2.** 33HPP concentrations in the plasma and in the cecal content of donor and recipient mice. (a) Plasma concentrations of 3-(3’-Hydroxyphenyl)propanoic acid (33HPP) in lean (Lean_do) and diet-induced obese donors (DIO_do) and their gut microbiota recipient mice (Lean_rec and DIO_rec respectively). (b) Cecal concentrations of 33HPP in lean (Lean_do) and diet-induced obese donors (DIO_do) and their gut microbiota recipient mice (Lean_rec and DIO_rec respectively). (c) PFC concentrations of 3-(Phenyl)propanoic acid-3′-sulfate (33HPP-S) in lean (Lean_do) and diet-induced obese donors (DIO_do) and their gut microbiota recipient mice (Lean_rec and DIO_rec respectively).  Data are shown as mean ± SEM (*n*=7-8/group). *p*-values were obtained after One-way ANOVA followed by Tukey post-hoc test. * : *p*-value ≤ 0,05; ** : *p*-value ≤ 0,01; *** : *p*-value≤ 0,001.

**Additional file 3.** Genus correlating significantly with the concentration of 33HPP in the plasma and in the cecum after Spearman’s correlation and False Discovery Rate (FDR).

**Additional file 4.** Correlations between 33HPP concentrations in the plasma and in the cecal content and abundance of bacteria in donor and recipient mice. Spearman’s correlations between 3-(3’-Hydroxyphenyl)propanoic acid (33HPP) concentrations in the plasma or in the cecal content of donor and gut microbiota recipient mice and the relative abundance of (a) *Akkermansia, *(b) *Muribaculum, *(c) Unknown *Prevotellaceae,*(d) *Alloprevotella*, and (e) *Parabacteroides *in lean (Lean_do) and diet-induced obese donors (DIO_do) and their gut microbiota recipient mice (Lean_rec and DIO_rec respectively).

**Additional file 5.** Sequences of primers for qPCR.

**Additional file 6.** Raw peak area for metabolomic analysis in the cecal content of lean (LD) and diet-induced obese donors (OD) and their gut microbiota recipient mice (OR and DR).

**Additional file 7.** Raw peak area for metabolomic analysis in the plasma of lean (LD) and diet-induced obese donors (OD) and their gut microbiota recipient mice (OR and DR).

## Data Availability

The datasets supporting the conclusions of this article are included within the article (and its additional files) or available in the European Nucleotide Archive (ENA/EBI) repository, https://www.ebi.ac.uk/ena/browser/view/PRJEB52720.

## Abbreviations

ASV: Amplicon sequence variant
CT: Control diet
CPP: Conditioned place preference
*Drd1*: Dopamine receptor 1
*Drd2*: Dopamine receptor 2
DAT: Dopamine transporter
FMT: Fecal material transplantation
FR: Progressive ratios sessions
GLP-1: Glucagon-like peptide 1
HFD: High-fat diet
HFHS: High-fat high-sucrose
33HPP: 3-(3′-Hydroxyphenyl)propanoic acid
34HPP: 3-(4’- Hydroxyphenyl)propanoic acid
33HPP-S: 3-(Phenyl)propanoic acid-3′-sulfate
NAc: Nucleus accumbens
*Oprd*: δ-opioid receptor
*Oprk*: κ-opioid receptor
*Oprm*: μ-opioid receptor
PCoA: Principal coordinates analysis
*Pdyn*: Pre-prodynorphin
PFC: Prefrontal cortex
PR: Progressive ratios sessions
PYY: Peptide YY
SCFAs: Short-chain fatty acids
TH: Tyrosine hydroxylase
VTA: Ventral tegmental area
