# Supplementary figures and images for: Obese-associated gut microbes and derived phenolic metabolite as mediators of excessive motivation for food reward
Source: Microbiome. 2023 Apr 28;11:94. doi: 10.1186/s40168-023-01526-w (PMC10142783; doi:10.1186/s40168-023-01526-w)

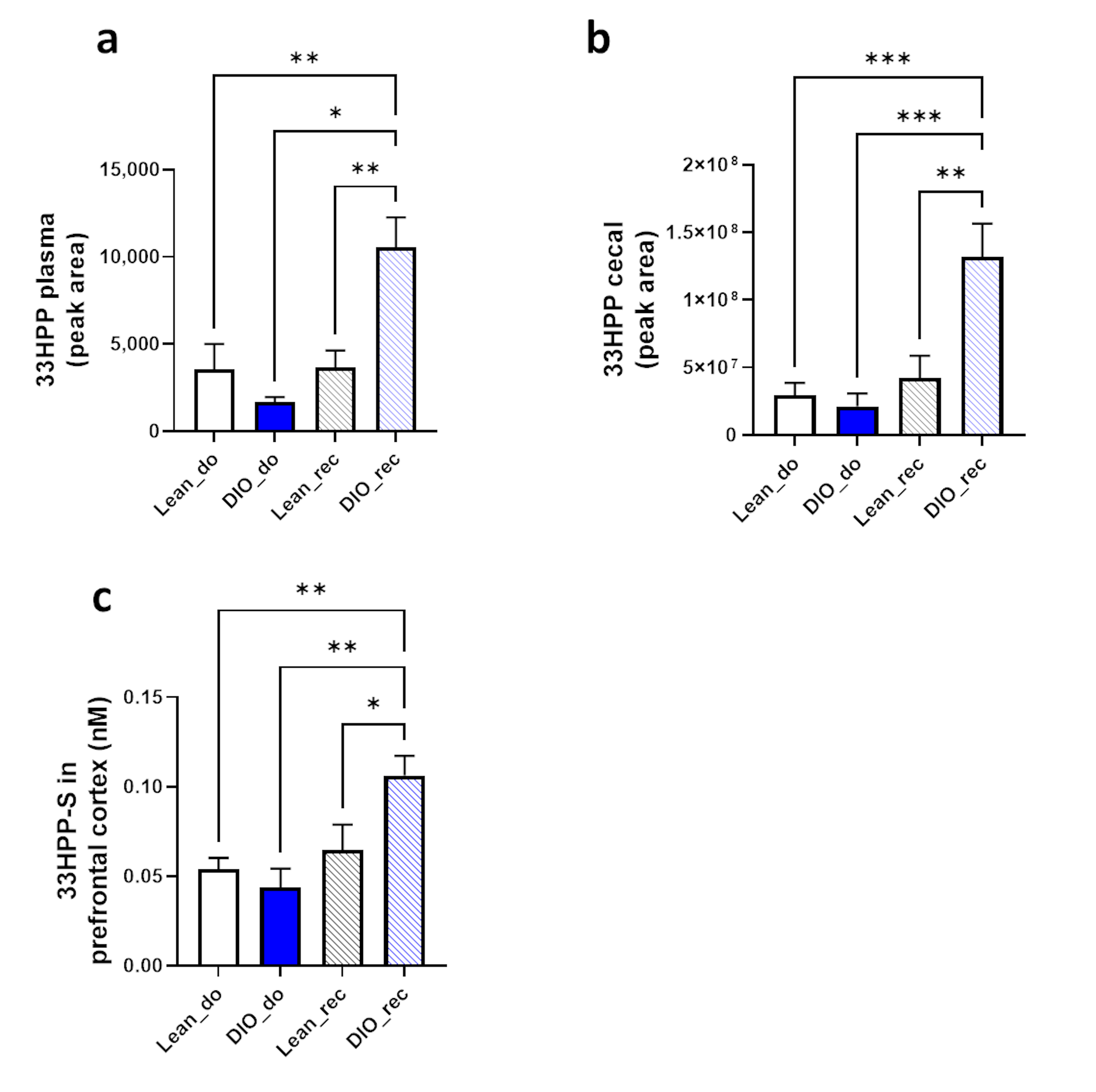

Supplement: Supplementary file 3 — Additional file 2. 33HPP concentrations in the plasma and in the cecal content of donor and recipient mice. (a) Plasma concentrations of 3-(3’-Hydroxyphenyl)propanoic acid (33HPP) in lean (Lean_do) and diet-induced obese donors (DIO_do) and their gut microbiota recipient mice (Lean_rec and DIO_rec respectively). (b) Cecal concentrations of 33HPP in lean (Lean_do) and diet-induced obese donors (DIO_do) and their gut microbiota recipient mice (Lean_rec and DIO_rec respectively). (c) PFC concentrations of 3-(Phenyl)propanoic acid-3′-sulfate (33HPP-S) in lean (Lean_do) and diet-induced obese donors (DIO_do) and their gut microbiota recipient mice (Lean_rec and DIO_rec respectively). Data are shown as mean ± SEM (n=7-8/group). p-values were obtained after One-way ANOVA followed by Tukey post-hoc test. * : p-value ≤ 0,05; ** : p-value ≤ 0,01; *** : p-value≤ 0,001. [file 40168_2023_1526_MOESM2_ESM.tiff]

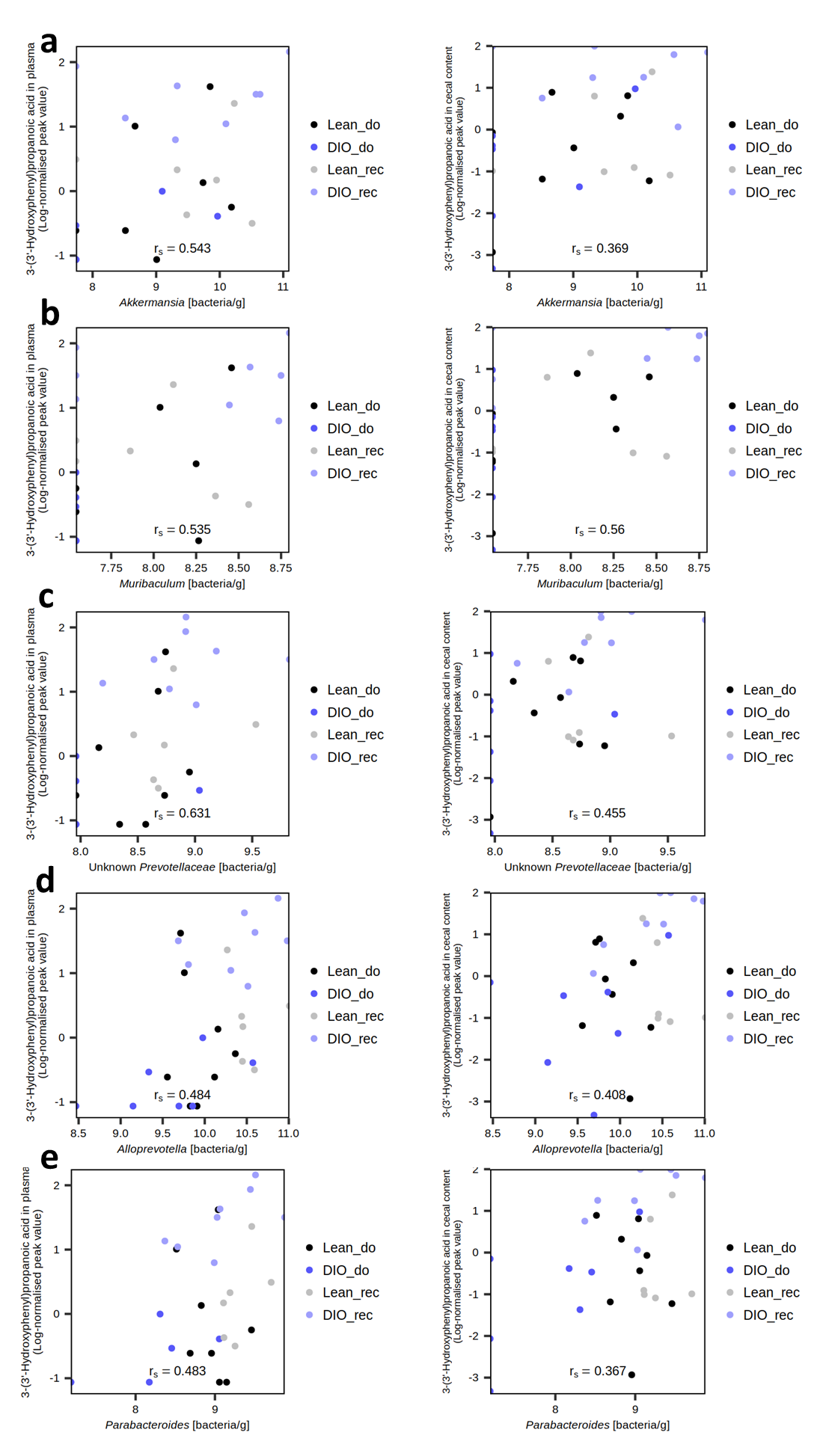

Supplement: Supplementary file 5 — Additional file 4. Correlations between 33HPP concentrations in the plasma and in the cecal content and abundance of bacteria in donor and recipient mice. Spearman’s correlations between 3-(3’-Hydroxyphenyl)propanoic acid (33HPP) concentrations in the plasma or in the cecal content of donor and gut microbiota recipient mice and the relative abundance of (a) Akkermansia, (b) Muribaculum, (c) Unknown Prevotellaceae,(d) Alloprevotella, and (e) Parabacteroides in lean (Lean_do) and diet-induced obese donors (DIO_do) and their gut microbiota recipient mice (Lean_rec and DIO_rec respectively). [file 40168_2023_1526_MOESM4_ESM.tiff]
